# Adipose-Derived Mesenchymal Stem Cell Application Combined With Fibrin Matrix Promotes Structural and Functional Recovery Following Spinal Cord Injury in Rats

**DOI:** 10.3389/fphar.2018.00343

**Published:** 2018-04-10

**Authors:** Yana O. Mukhamedshina, Elvira R. Akhmetzyanova, Alexander A. Kostennikov, Elena Y. Zakirova, Luisa R. Galieva, Ekaterina E. Garanina, Alexander A. Rogozin, Andrey P. Kiassov, Albert A. Rizvanov

**Affiliations:** ^1^OpenLab “Gene and Cell Technologies”, Institute of Fundamental Medicine and Biology, Kazan Federal University, Kazan, Russia; ^2^Department of Histology, Cytology and Embryology, Kazan State Medical University, Kazan, Russia; ^3^Department of Neurology and Manual Therapy, Kazan State Medical Academy, Kazan, Russia

**Keywords:** spinal cord injury, mesenchymal stem cells, adipose tissue, fibrin matrix, rats

## Abstract

The use of stem and progenitor cells to restore damaged organs and tissues, in particular, the central nervous system, is currently considered a most promising therapy in regenerative medicine. At the same time, another approach aimed at stimulating regeneration with the use of stem cells encapsulated into a biopolymer matrix and capable of creating a specific microenvironment for the implanted cells similar to the natural extracellular matrix is under active development. Here, we study effects of the application of adipose-derived mesenchymal stem cells (AD-MSCs) combined with a fibrin matrix on post-traumatic reactions in the spinal cord in rats. The AD-MSC application is found to exert a positive impact on the functional and structural recovery after spinal cord injury (SCI) that has been confirmed by the results of behavioral/electrophysiological and morphometric studies demonstrating reduced area of abnormal cavities and enhanced tissue retention in the site of injury. Immunohistochemical and real-time PCR analyses provide evidence that AD-MSC application decreases the GFAP expression in the area of SCI that might indicate the reduction of astroglial activation. Our results also demonstrate that AD-MSC application contributes to marked upregulation of PDGFβR and HSPA1b mRNA expression and decrease of Iba1 expression at the site of the central canal. Thus, the application of AD-MSCs combined with fibrin matrix at the site of SCI during the subacute period can stimulate important mechanisms of nervous tissue regeneration and should be further developed for clinical applications.

## Introduction

Thousands of people worldwide suffer from the consequences of spinal cord injury (SCI). SCI results in impaired sensory and motor functions unavoidably leading to severe disability. The development of new therapeutic approaches to the treatment of SCI which enable functional recovery is urgently required and it would promote clinical medicine significantly. At present regenerative medicine seems to be quite promising; when aimed at maintaining natural healing in tissue and organ reconstruction, providing a supportive environment where tissue damage or loss can be completely restored (Vacanti et al., 2014). The approaches of regenerative medicine developed to date include stem and progenitor cell transplantation, manipulations using the patient’s own cells and the use of scaffolds.

The approach aimed at stimulating regeneration of injured spinal cord with the use of stem cells encapsulated in biopolymer matrix is under active development (Zeng et al., 2011, 2016; Assunção-Silva et al., 2015; Carlson et al., 2016; Caron et al., 2016; Zhao et al., 2017). These technologies are used to maintain matrix-encapsulated cells, to target their differentiation, and to create a specific environment for them which is similar to a natural extracellular matrix. However, systemic, intraspinal and intrathecal routes of administration have significant disadvantages associated with limited quantities and a low viability of stem cells *in situ*.

The potential of matrices is most often evaluated by their ability to maintain the viability of cells enclosed within them for a short period of time *in vitro* (Novikova et al., 2006; Pedraza et al., 2009; Ribeiro-Samy et al., 2013). To date several studies have revealed the advantages using stem cells encapsulated in biopolymer matrices after SCI *in vivo.* For example, an intraspinal injection of neural stem cells combined with a hydrogel based on hyaluronic acid and methyl cellulose and covalently-modified with recombinant rat platelet-derived growth factor-A into an area of spinal cord compression in rats was shown to improve the motor function and viability of the engrafted cells, reduce abnormal cavitation, enhance the differentiation of oligodendrocytes and to promote neuron surviveal (Mothe et al., 2013). The embryonic stem cell-derived neural progenitor cells transplanted within fibrin scaffolds enhance functional recovery in a subacute model of dorsal hemisection lesion SCI (Johnson et al., 2010). Implantation of Schwann cells as cell suspensions with *in situ* gelling laminin:collagen matrices in the subacute period of SCI significantly enhances long-term cell survival, improves graft vascularization as well as the degree of axonal in-growth (Patel et al., 2010).

Nevertheless, mesenchymal stem cells (MSCs) elicit the greatest interest in the clinical use. The neuroregeneratory potential of MSCs is due to the following positive properties of these cells: (1) the possibility of secretion of various neurotrophic factors and cytokines, (2) the possibility of trans-differentiation into cells of non-mesenchymal origin, including neurons and glial cells, and (3) immunomodulatory, anti-apoptotic, and anti-inflammatory effects (3–5) (Cui et al., 2013; Laroni et al., 2015; Masgutov et al., 2016; Lo Furno et al., 2017). Therefore, there are numerous studies of the therapeutic potential of combinatorial approaches based on MSC therapy and biomaterials for SCI treatment. The transplantation of bone marrow-MSCs combined with a gelatin matrix into the area of complete rat spinal cord transection in the subacute period improves inflammation, stimulates angiogenesis, reduces abnormal cavitation (Zeng et al., 2011) and promotes regeneration of nerve fibers (Zeng et al., 2016). Caron et al. (2016) implanted human umbilical cord blood-derived MSCs combined with hydrogel into the area of injury immediately after moderate compression of the lower thoracic spine in mice. They demonstrated that this type of treatment can significantly modify the immune response in a proinflammatory environment within the area of SCI by increasing the macrophage M2 population and promoting an appropriate microenvironment for regeneration (Caron et al., 2016). Despite the significant number of similar studies for the successful translation of the results to the clinic, it is necessary to consider the following seven aspects: adequacy of the pre-clinical SCI model, time (the post-traumatic period) and the method of delivery of MSCs embedded in matrices (1–3), the optimal choice the design of a biomaterial and its applicability in routine neurosurgery (4), study of the main links in the pathogenesis of SCI (astroglial activation, inflammation, activation of microglia), as well as structure (morphometry) and function (behavioral and electrophysiological studies) of injured spinal cord after used combinatorial approaches in treatment (5–7).

We have studied the effects of the application of adipose-derived mesenchymal stem cells (AD-MSCs) combined with a fibrin matrix on structural and functional recovery following SCI in a subacute period in rats, as much as possible satisfying the criteria noted above. Our results demonstrated that the AD-MSC application is found to exert a positive impact on the functional and structural recovery after SCI that has been confirmed by the behavioral/electrophysiological and morphometric studies demonstrating reduced area of abnormal cavities and enhanced tissue retention in the site of injury. Immunohistochemical and real-time PCR analyses provide evidence that AD-MSC application decreases the GFAP and Iba1 expression in the area of SCI. We also observed that the AD-MSC application contributes to markedly upregulation of PDGFβR and HSPA1b mRNA expression.

## Materials and Methods

### Isolation and Preparation of Rat Mesenchymal Stem Cells

Adipose-derived mesenchymal stem cells were derived from female Wistar rats (weighting 250–300 g, *n* = 5, Pushchino Laboratory, Russia) as previously described (Mukhamedshina et al., 2017b). The adipose tissue was cut into pieces of about 1 mm^3^. Blood cells were washed out by centrifugation at 500 *g* for 5 min. The adipose tissue was incubated with crab hepatopancreas collagenase (Biolot, Russia) solution at a final concentration of 0.2% for an hour at 37°C with shaking at 200 rpm. The homogenate was centrifuged for 5 min at 500 *g*, the enzymatic solution decanted. The cell pellet containing the stromal-vascular fraction was suspended with Dulbecco’s phosphate-buffered saline solution and centrifuged for 5 min at 500 *g* twice to remove residual enzymes. The cells were cultured in minimum essential medium α with 10% fetal bovine serum, 100 U/ml penicillin, 100 μg/ml streptomycin and 2 mM L-glutamine (all obtained from PanEco, Russia). The culture medium was changed every 3 days. The third passage cells were used for immunophenotyping and experiments. Previously we reported that cells obtained in this manner had fibroblast-like morphology, adhered to the bottom of a culture flask and expressed Thy-1, CD 73, Stro-1 and CD 44, which satisfies minimum compliance criteria peculiar for MSCs (Mukhamedshina et al., 2017b).

The AD-MSCs were transduced with lentiviral vectors encoding enhanced green fluorescent protein (EGFP) (AD-MSCs+LV-EGFP) as previously described for subsequent transplantation in the area of SCI (Solovyeva et al., 2015). Briefly, LV-EGFP viral particles were produced in HEK293FT cell line after calcium-phosphate co-transfection with vector plasmid pWPT-GFP (12255, AddGene; 10, 14 μg per 10 cm-well plate), packaging plasmid psPAX2 (12259, AddGene; 6, 63 μg per 10 cm-well plate) and envelope plasmid pCMV-VSV-G (8454, AddGene; 3, 52 μg per 10 cm-well plate). Viral particles were concentrated from supernatant using ultracentrifugation with 26,000 rpm for 2 h at +4°C (Optima L-90K, Beckman Coulter) and stored at -80°C. The percentages of EGFP-positive cells were assessed by flow cytometry (Guava EasyCyte 8HT, Millipore, United States). Previously we reported that after viral transduction, AD-MSCs typically begin to express EGFP within 48 h, with a plateau reached in 96 h and seventy percent of the cells in the population studied were EGFP-positive (Mukhamedshina et al., 2017b).

### Spinal Cord Contused Injury and Cell Application

All animal protocols were approved by the Kazan Federal University Local Ethical Committee (Protocol NO. 2, May 5^th^ 2015). Twenty rats were assigned randomly into the experimental and control groups (**Table 1**) and were housed in clear plastic cages (12 h:12 h light/dark cycle) with food and water available *ad libitum*.

**Table 1:** Investigated groups.

Groups of animals	Duration of experiment, days	Methods	Number of animals
Intact control	–	RT-PCR	5
	–	Immunohistochemistry	5
	–	Electrophysiological studies	10
SCI Tissucol (control group)	74	Immunohistochemistry, Histology	5
	74	RT-PCR	5
	74	Electrophysiological studies, BBB	15
SCI Tissucol+AD-MSCs+LV-EGFP (experimental group)	74	Immunohistochemistry, Histology	5
	74	RT-PCR	5
	74	Electrophysiological studies, BBB	15


Spinal cord contused injury and cell application were carried out as previously described (Shaymardanova et al., 2012). Rats were deeply anesthetized with an intraperitoneal injection of chloral hydrate (80 mg/ml, 0.4 ml per 100 g, Sigma). After skin incision, laminectomy at the Th8 vertebral level was performed. The impact rod (2 mm diameter, 10 g) of an impactor was centered above Th8 and dropped from 25 mm height to induce SCI as previously described (Mukhamedshina et al., 2016). After SCI the dorsal back muscles and the skin were sutured. After surgery the rats had intramuscular injections of gentamicin (25 mg/kg, Omela, Russia) for 7 consecutive days. The injured rats’ bladders were manually emptied twice daily until spontaneous voiding occurred.

The skin was re-incised to expose the spinal cord 2 weeks after SCI. Then, AD-MSCs+LV-EGFP (1 × 10^6^ cells) mixed with Tissucol fibrin sealant (18 μL, Baxter) were applied on top of the injury (Tissucol+AD-MSCs+LV-EGFP or experimental group, *n* = 10). The control group received Tissucol applied onto the above mentioned area (Tissucol group, *n* = 10). Then the wound was sutured. After surgery, the rats received daily intramuscular doses of gentamicin (25 mg/kg, Omela, Russia) for 7 consecutive days.

### Behavioral Study

Motor function was evaluated using the open-field Basso, Beattie, Bresnahan (BBB) locomotor rating scale. A baseline was obtained 3 days before SCI. To evaluate differences in functional recovery, a behavioral assessment in the control and experimental groups was performed before SCI, on day 1 and once a week from 1 to 11 weeks after initial SCI surgery. Locomotion was scored simultaneously by 2 observers who were blinded to the treatment groups. Final scores were obtained by averaging the two scores awarded by the examiners.

### Electrophysiological Studies

Electrophysiological tests were performed on intact rats and control/experimental rats 2 and 11 weeks after SCI as previously described (Mukhamedshina et al., 2017a). The animal’s neuromotor function was assessed by stimulating electromyography. The M- and H-waves from the gastrocnemius muscle were recorded in response to stimulation of the sciatic nerve. Monopolar needle electrodes were used for both recording and reference. An active electrode was inserted into the middle of the muscle belly, with the reference one implanted within a region of the tendomuscular junction at the Achilles tendon. Electrical stimulation of the sciatic nerve was carried out with square-wave single stimuli lasting for 0.2 ms. Monopolar needle electrodes inserted subcutaneously within an area where the sciatic nerve exits from the pelvis were used for stimulation.

Transcranial electrical stimulation was used for evaluation of pyramidal tracts. Motor evoked potentials (MEPs) were registered from gastrocnemius muscle by the same technic as M-response. Transcranial stimulation was performed by needle electrodes inserted under the scalp up to the contact with the bone of the skull. Cathode was placed in the middle approximately 0.5 cm caudally from the interorbital line. Anode was placed in the middle near the occipital bone. Stimuli 0.04 ms with intensity from 20 to 400 V were used.

Somatosensory evoked potentials (SEPs) were used for evaluation of posterior columns of spinal cord. Monopolar needle electrodes inserted subcutaneously were used for registration. For registration from lumbar level active electrode was inserted over upper lumbar vertebras, referent electrode – over middle thoracic vertebras. For registration from scalp active electrode was inserted in the middle approximately 0.5 cm caudally from the interorbital line, referent electrode – in the middle near the occipital bone. Electrical stimulation of tail performed by round electrodes with stimulus duration 0.2 ms. Stimulus intensity was chosen by the tail movements (smallest stimulus producing tail movements was used).

### Histological Assessment

For histology and immunohistochemistry, 60 days after cell application rats were anesthetized with chloral hydrate and subjected to intracardiac perfusion with 4% paraformaldehyde (PFA, Sigma). After incubation in 30% sucrose, the samples were embedded in a tissue freezing medium (Tissue-Tek^®^ O.C.T. Compound, Sakura^®^ Finetek). Non-fixed tissue was used for RT-PCR. Twenty micrometer transverse tissue sections, obtained with a Microm HM 560 cryostat, were stained with Azur-eosin to visualize tissues. Images were captured using 20x objective lens and microscope (APERIOCS2, Leica). The cross-sectional area of the spared tissue and abnormal cavities was measured on transverse sections of the spinal cord within the midpoint of the lesion center (the epicenter) and in spinal segments 1–5 mm rostral and caudal to the site of injury. A total area of abnormal cavities in the spinal cord cross-section was calculated by adding cysts with an area of not less than 1.500 μm^2^. Aperio imagescope software was used to measure the tissue area.

To count the number of muscle fibers in the calf muscle, sections were stained with hematoxylin and eosin and the number of fibers counted over the entire area of a circle (0.125 mm^2^). 15 fibers were selected in this circle to calculate the value. Thus, a total of 150 fibers were examined in every group of rats. The resulting specimens were studied under Axio Observer Z.1 microscope (Carl Zeiss) using ImageJ Version 1.46 software.

### Immunohistochemical Studies

Transverse tissue sections were incubated with primary and secondary antibodies (Abs) shown in **Table 2**. For immunofluorescence labeling, the sections were blocked with 5% normal goat serum for 1 h at room temperature (RT) and then incubated overnight at 4°C with a primary Abs. Prior to visualization, the sections were incubated with corresponding fluorophore-conjugated secondary Abs for 2 h at RT. 4′,6-Diamidino-2-phenylindole (DAPI) (10 μg/mL in PBS, Sigma) to visualize nuclei. Coverslips were mounted on slides using mounting medium (ImmunoHistoMount, Santa Cruz). The sections were examined using a LSM 780 Confocal Microscope (Carl Zeiss, Germany). The mean intensity of labeling (semi-quantitative analysis of GFAP and Iba1) was analyzed using Zen 2012 Software (Carl Zeiss). All sections were imaged in the z-plane using identical confocal settings (laser intensity, gain and offset). Measurements were obtained from transverse histological sections collected at 5-mm increments extending from the contusion center of the SCI. The following areas were selected for a semiquantitative immunohistochemical evaluation of glial cells: the ventral horn (VH), the main corticospinal tract (CST), ventral funiculi (VF), the area around the central canal (CC) and the dorsal root entry zone (DREZ).

**Table 2:** Primary and secondary antibodies used in immunofluorescent staining.

Antibody	Host	Dilution	Source
GFAP	Mouse	1:200	Millipore, MAB360
Iba1	Goat	1:300	Abcam, ab5076
Anti- goat IgG conjugated with Alexa 555	Donkey	1:200	Invitrogen, A21432
Anti- mouse IgG conjugated with Alexa 546	Donkey	1:200	Invitrogen, A11003


### RNA Isolation, cDNA Synthesis, and Real-Time PCR

Total RNA was isolated from fresh spinal cords (5 mm long segment encompassing the injury site) using Yellow Solve Kit (Silex, Russia) according to the manufacturer’s recommendations. The first strand cDNA synthesis was performed using 100 ng of total RNA, 100 U of RevertAid reverse transcriptase (Thermo Fisher Scientific), 100 pmol of random hexamer primers and 5 U of RNAse inhibitor according to the recommended manufacturer’s protocol (25°C–10 min, 42°C–60 min, termination of transcription 70°C–10 min) A quantitative analysis of mRNA of *Gfap*, *Vimentin*, *S100*, *Pmp22*, *Pdgf*α, *Pdgf*β, *Vegf*, *Fgf2* genes was performed using CFX 96 Real-Time PCR System (Bio-Rad, Hercules, CA, United States). Amplification procedure was performed as following: 95°C–3 min, 39 cycles: 95°C–10 s, 55°C–30 s including plate read. Each PCR reaction contained 100 ng of preliminarily diluted cDNA, 2.5× Reaction mixture B (Syntol, Russia), 200 nM of each primer and 100 nM probe (**Table 3**). The mRNA expression was normalized using 18S rRNA. Plasmid DNA with corresponding inserts was used to build a standard curve. To assess copy numbers of plasmid DNA insert we used DNA copy number calculator^1^. The mRNA level in intact spinal cord at Th8 level was considered as 100%. Cq range for 18S was 13–14 cycles, for other genes: 25–31. Compatibility of fluorescent dye and quenchers was confirmed with manuals of Syntol^2^ and Thermoscientific^3^ companies.

**Table 3:** Primers and probes for RT-PCR.

Primer	Accession number	Nucleotide sequence
18S-TM-Forward	NM_001135743.1	gCCgCTAgAggTgAAATTCTTg
18S-TM-Reverse	NM_001135743.1	CATTCTTggCAAATgCTTTCg
18S-TM-Probe	NM_001135743.1	[HEX]ACCgCgCAAgACggACCAg[BH2]
V164-TM-Forward	NM_001110334.2, NM_001287107.1	TATATCTTCAAgCCgTCCTgTg
V164-TM-Reverse	NM_001110334.2, NM_001287107.1	TCTCCTATgTgCTggCTTTg
V164-TM-Probe	NM_001110334.2, NM_001287107.1	[FAM]TCCgCATgATCTgCATAgTgACgTTg[BH2]
Vim-TM-Forward	NM_031140.1	ACCCTgCAgTCATTCAgACA
Vim-TM-Reverse	NM_031140.1	TCCTggATCTCTTCATCgTg
Vim-TM-Probe	NM_031140.1	[HEX] CTggCACgTCTTgACCTTgAACg [BH2]
S100b-TM-Forward	NM_013191.1	GAgAgAgggTgACAAgCACA
S100b-TM-Reverse	NM_013191.1	CACCACTTCCTgCTCTTTgA
S100b-TM-Probe	NM_013191.1	[FAM] CgAgCTCTCTCACTTCCTggAggAA [BH1]
Pmp22-TM-Forward	NM_017037.1	gTgCTAgTgTTgCTCTTC
Pmp22-TM-Reverse	NM_017037.1	GgATgTggTACAgTTCTg
Pmp22-TM-Probe	NM_017037.1	[FAM] CTCCACCATCgTCAgCCAAT [BH1]
PDGFRa-TM-Forward	NM_012802.1	GgTTAgAggAgCACCTggAg
PDGFRa-TM-Reverse	NM_012802.1	TCTCACCTCACATCCgTCTC
PDGFRa -TM-Probe	NM_012802.1	[FAM] ATgCgCgACCTCCAACCTgA [BH1]
PDGFb-TM-Forward	NM_031525.1	CTgCAATAACCgCAATTgTg
PDGFb-TM-Reverse	NM_031525.1	TCgATCTTTCTCACCTgCAC
PDGFb-TM-Probe	NM_031525.1	[FAM] CCgCATCTgCACCTgCgAg [BH1]
FGF2-TM-Forward	NM_019305.2	GCTgCTggCTTCTAAgTgTg
FGF2-TM-Reverse	NM_019305.2	GTgCCACATACCAACTggAg
FGF2-TM-Probe	NM_019305.2	[FAM] TCTTCTTTgAACgCCTggAgTCCA [BH1]
Iba1-TM-Forward	NM_017196.3	ACCAGCGTCTGAGGAGCTAT
Iba1-TM-Reverse	NM_017196.3	AGGAAGTGCTTGTTGATCCC
Iba1-TM-Probe	NM_017196.3	[HEX]CCCTGCAAATCCTTGCTCTGGC[BH2]


### Statistical Analysis

Data are presented as mean ± standard deviation (SD). A Student’s *t*-test, a one-way analysis of variance (ANOVA) with Tukey’s test or two-way analysis of variance (ANOVA) were used for multiple comparisons between all investigated groups. All analyses were performed in a blinded manner with respect to the treatment group. A value of *P* < 0.05 was considered statistically significant. Data were analyzed using Origin 7.0 SR0 Software (OriginLab, Northampton, MA, United States).

## Results

### Assessment of Locomotor Activity

**FIGURE 1:**
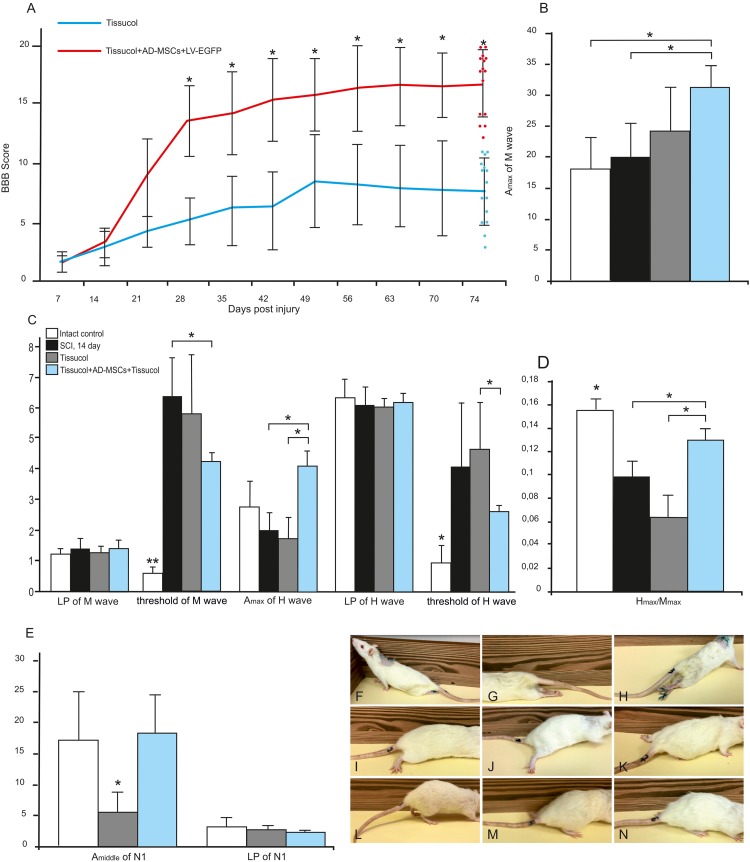
Behavioral nd electrophysiological studies after SCI in control and experimental groups. BBB locomotor scores of rats of the Tissucol (blue line) and Tissucol+AD-MSCs+LV-EGFP (red line) groups. The motor function scores were higher in Tissucol+AD-MSCs+LV-EGFP group than those in the Tissucol group on week 11 after injury, with significant differences for at least within 8 post-injury weeks. ^∗^*P* < 0.05, one-way ANOVA followed by a Tukey’s *post hoc* test **(A)**. Amax of M-wave **(B)**, Amax of H-wave, LP and a threshold of M- and H-waves **(C)**, H/M wave amplitude ratio **(D)** after SCI in the experimental groups. ^∗^*P* < 0.05, ^∗∗^*P* < 0.01, one-way ANOVA followed by a Tukey’s *post hoc* test. Middle amplitude and LP of lumbar N1 after SCI in the experimental groups **(E)**. Video images of a spinal cord-injured rats showing absent or slight movements at day 7 after SCI **(F–H)**, extensive movement of joints with no weight support steeping **(I,J)** and occasional dorsal stepping **(K)** in Tissucol group at day 74 after SCI, two step cycles with weight supported plantar stepping and coordination between the forelimb/hindlimb and tail consistently up in Tissucol+AD-MSCs+LV-EGFP group at day 74 after SCI **(L–N)**.

We assessed locomotor recovery using the BBB rating scale from 1 to 11 weeks after injury. The motor function scores were higher in the Tissucol+AD-MSCs+LV-EGFP group (17.1 ± 3.1) than those in the Tissucol-treated group (6.7 ± 2.5) on week 11 after injury (**Figure 1A**), with significant differences (*P* < 0.05) within 8 post-injury weeks. Thus, the behavioral data from the BBB locomotor scores demonstrate that the application of Tissucol+AD-MSCs+LV-EGFP after SCI had dramatically improved the neurological function (**Figures 1F–N**).

**FIGURE 2:**
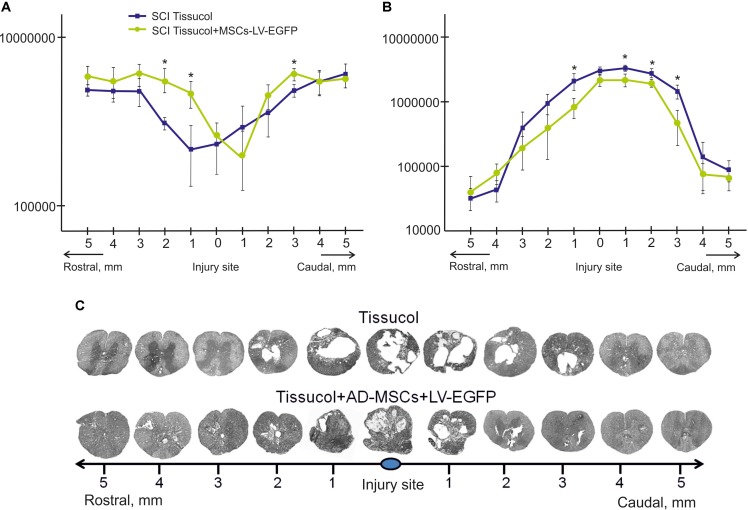
Tissue analysis in control and experimental groups. An area of the spared tissue **(A)** and a total area of abnormal cavities **(B)** 5 mm rostrally and caudally from the injury epicenter in 60 days after Tissucol (blue line) or Tissucol+AD-MSCs+LV-EGFP (yellow line) application. ^∗^*P* < 0.05, one-way ANOVA followed by a Tukey’s *post hoc* test. Cross-sections of the injured spinal cord 60 days after Tissucol or Tissucol+AD-MSCs+LV-EGFP application 5 mm rostrally and caudally from the injury epicenter **(C)**. Azur-eosin staining.

### Electrophysiology Results

On day 14 there were no differences in the Ì-wave amplitude with the intact group suggesting that SCI at the Th8 level did not result in significant damage to the lumbar neurons. On day 60 the amplitude of M-wave increased in the experimental group (Tissucol+AD-MSCs+LV-EGFP) as compared to the intact control and the Tissucol groups without cell application (**Figure 1B**). Given that the M-wave amplitude remains on post-injury day 14 this change seems unexpected. A rise of the M-wave amplitude might be a marker of the increased number of functionally active fibers in a muscle and indirectly reflect an augmentation of the axon number within the nerve examined. We hypothesize that the application of AD-MSCs can increase the number of muscle fibers and performed the morphometry of the calf muscles of the studied animals. However, the study did not reveal any significant changes in the number of muscle fibers between the control and experimental groups (data not shown).

There was also no difference in the latency of H-wave between the control/experimental and intact groups on day 14, which suggests the retention of the H-reflex arc (**Figure 1C**). The H-wave latency remained unchanged by day 60 in all groups studied. At the same time, there was a decreased ratio of the H-wave to M-wave amplitude that might reflect a reduced reflex excitability of lumbar neurons in response to injury. On day 60 there was progress toward restoring the H/M-wave amplitude ratio in the group with AD-MSC application (**Figure 1D**). Thus, neurophysiological parameters suggest the application of Tissucol-encapsulated AD-MSCs is superior to that of Tissucol without cells.

Transcranial electrical stimulation in intact rats lead to registration of MEPs form gastrocnemius muscle with amplitude 17.66 ± 5.30 mV, and latency 4.68 ± 0.53 ms. In the SCI Tissucol group MEPs were not registered from both legs in 42.8% cases and in 14.2% cases MEP was registered from one side. Middle amplitude of registered MEPs in the SCI Tissucol group was 0.5 ± 0.56 mV, latency 15.83 ± 1.14 ms. In the SCI Tissucol+AD-MSCs-LV-EGFP group MEPs were not registered from both sides in 20% cases and in 20% cases MEPs were registered from one side. Middle amplitude of registered MEPs in the SCI Tissucol+AD-MSCs-LV-EGFP group was 1.57 ± 0.88 mV, latency 14.08 ± 3.83 ms.

Somatosensory evoked potentials in intact rats from the lumbar level were presented by P1- N1-P2 peaks. Peak N1 was most prominent and N1 amplitude and latency were evaluated. Middle amplitude of lumbar N1 was 16.72 ± 8.68 μV, latency 3.08 ± 0.97 ms (**Figure 1E**). Scalp electrodes registered potential looked as P1–N1 complex. Amplitude and latency of P1 were evaluated. Middle amplitude of scalp P1 was 7.87 ± 2.60 μV, latency 9.31 ± 1.68 ms in intact rats.

Apart of the results from transcranial electrical stimulation practically no recovery of scalp SEPs was noted. Scalp SEP was registered only in 20% cases from SCI Tissucol+AD-MSCs-LV-EGFP group and wasn’t registered in SCI Tissucol group. Lumbar peaks were preserved in both groups but amplitude of lumbar N1 peak was signficantly lower in SCI Tissucol group comparing with intact and SCI Tissucol+AD-MSCs-LV-EGFP groups. Most probable generators of lumbar peaks are spinal roots and neurons of dorsal horns of the lumbar and sacral segments of spinal cord. We can suggest that spinal trauma on the thoracic level lead to edema and secondary disturbed blood circulation in the lower segments of spinal cord also, what could cause damage of spinal roots or neurons of dorsal horns. Lack of changes of lumbar N1 amplitude comparing with intact group could be a result of AD-MSCs-LV-EGFP application.

### Tissue Sparings

The area of the spared tissue was larger in the rostral and in part caudal direction from the injury epicenter in the experimental group of rats 60 days after the application of Tissucol+AD-MSCs+LV-EGFP in the area of SCI as compared to the control group (Tissucol only). At the same time, there was a statistically significant difference (*P* < 0.05) in spared tissue area between the experimental and control groups:

- 3 mm caudally from the injury epicenter, where this value is 1.23 times higher in the experimental group than in the control one;
- 1 and 2 mm rostrally from the epicenter of injury, where this value was 2.12 and 1.8 times higher in the experimental group than in the control group, respectively (**Figures 2A,C**).

The total area of abnormal cavities was also compared in the experimental groups. This parameter was found to be less 3 mm rostrally and up to 5 mm caudally from the epicenter of injury in the experimental group (Tissucol+AD-MSCs+LV-EGFP) in comparison with the control one (Tissucol only). There was a statistically significant difference (*P* < 0.05) in the total area of abnormal cavities between the experimental and control groups:

- 1, 2, and 3 mm caudally from the epicenter of injury, where this value was 32.7, 23.3, and 3.1% times less, respectively, between the experimental and control groups;
- 1 mm rostrally, where this value was 2.5 times less in the experimental group than in the controls (**Figures 2B,C**).

Thus, the application of AD-MSCs resulted in a reduced cavity volume and improved tissue retention in the subacute phase following SCI.

### Assessment of Astroglial Cells

**FIGURE 3:**
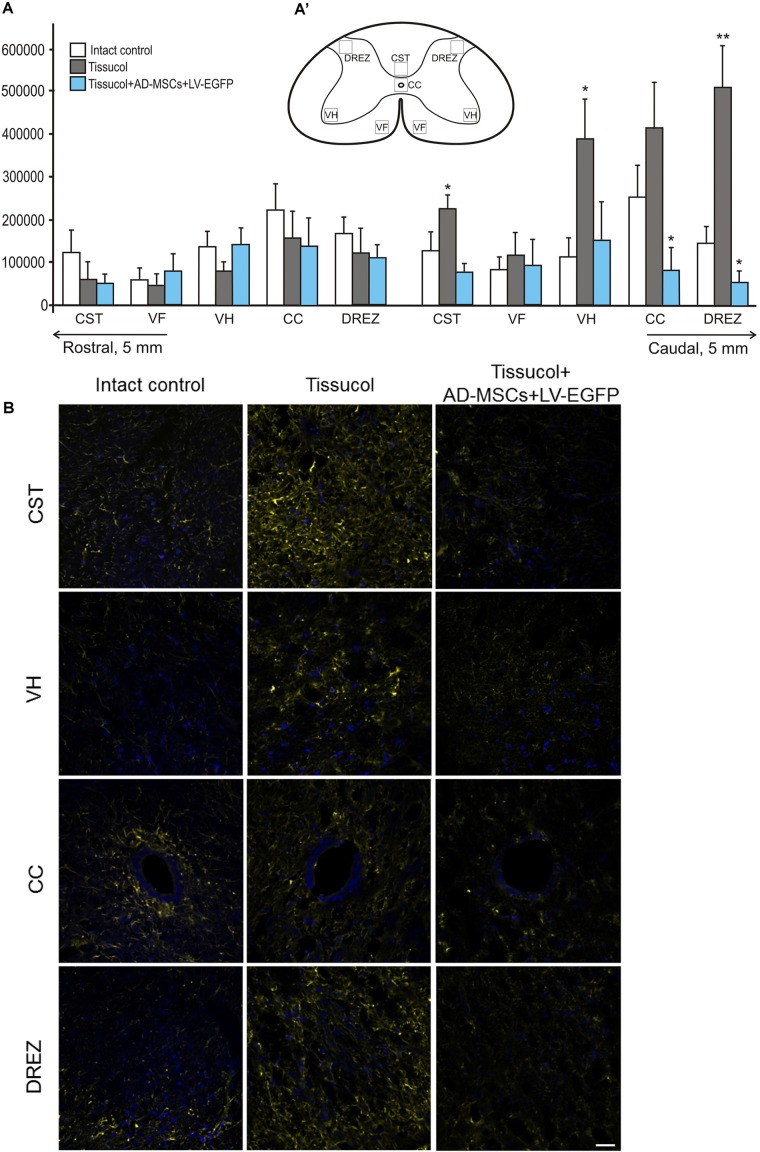
Astroglial activation at the site of injury. **(A)** The mean intensity of GFAP (Y axis) in intact spinal cord (write column) or 60 days after Tissucol (gray column) and Tissucol+AD-MSCs+LV-EGFP (blue column) application in the examined regions. ^∗^*P* < 0.05, ^∗∗^*P* < 0.01, one-way ANOVA followed by a Tukey’s *post hoc* test. **(A’)** In the rat spinal cord, five areas were selected for immunohistochemical evaluation: the ventral horn (VH), the main corticospinal tract (CST), the ventral funiculi (VF), the area of the central canal (CC) and the dorsal root entry zone (DREZ). **(B)** Visualization of astroglial activation using GFAP (yellow) 5 mm caudally from the injury epicenter within the CST, VH, CC, and DREZ in the investigated groups. Nuclei are DAPI-stained (blue). Scale bar: 25 μm.

Astroglial activation was assessed in spinal cords at a distance of 5 mm rostrally and caudally from the injury epicenter using an immunofluorescence assay with antibodies to GFAP (**Figures 3A,A’,B**). There were statistically significant differences between the investigated groups in the caudal direction from the injury epicenter, namely, within the regions of CST, VH, CC and the DREZ (**Figure 3A**). On day 60 after the application of Tissucol+AD-MSCs+LV-EGFP, there were lower levels of the GFAP expression relative to the intact controls in the CST, CC (*P* < 0.05), DREZ (*P* < 0.05), and Tissucol group in the CST (*P* < 0.05), VH (*P* < 0.05), CC (*P* < 0.05), and DREZ (*P* < 0.01). There was a more than 10-fold decrease of the GFAP level in the DREZ in the Tissucol+AD-MSCs+LV-EGFP group when compared with the Tissucol one. In the Tissucol only group the GFAP expression increased significantly in all examined regions in the caudal direction from the epicenter of injury as compared to the rostral direction from the site of injury. Thus, we have demonstrated that the application of Tissucol+AD-MSCs+LV-EGFP into the site of SCI in the subacute period reduced astroglial activation caudally from the injury epicenter.

### Assessment of Microglial Cells

**FIGURE 4:**
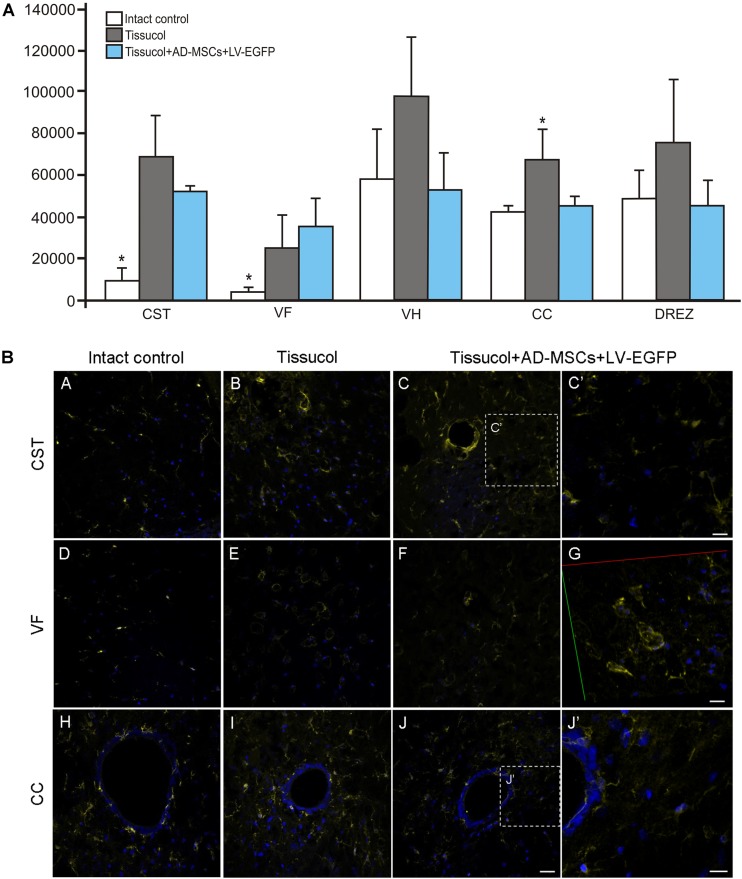
Assessment of microglial cells at the site of injury. **(A)** The mean intensity of Iba1 (Y axis) in intact spinal cord (write column) or 60 days after Tissucol (gray column) and Tissucol+AD-MSCs+LV-EGFP (blue column) application within the examined regions. ^∗^*P* < 0.05, one-way ANOVA followed by a Tukey’s *post hoc* test. **(B)** Assessment of microglial cells with Iba1 (yellow) 5 mm caudally from the injury epicenter within the CST, VF, and CC in the investigated groups. Nuclei are DAPI-stained (blue). Dashed boxed areas in C, J are shown in C′,J′. G show 3D confocal microscopy image in VF of Tissucol+AD-MSCs+LV-EGFP group. Scale bar: 25 (A–J) and 10 (C′,G,J′) μm.

Iba1 is a marker of microglial cells that detects both quiescent and reactive microglial cells (Chelyshev et al., 2014). In the investigated group, Iba1^+^ microglia homogeneously occupied every studied area in the spinal cord (**Figure 4B**). Nevertheless, the Iba1 expression was markedly upregulated after SCI at a distance of 5 mm caudal from the injury epicenter (**Figure 4A**). In the control and experimental groups, there was a more than fivefold increased Iba1 expression within the CST and VF as compared to the intact controls. However, there was a significant difference only within the CC zone, where the Iba1 expression was 56.3% lower (*P* < 0.05) in the Tissucol+AD-MSCs+LV-EGFP group as compared to the Tissucol group, and it actually did not differ from the same value the intact controls. Thus, we have demonstrated that the application of Tissucol+AD-MSCs+LV-EGFP into the site of SCI reduces the number of microglial cells in the area of CC.

### Analysis of Changes in the mRNAs Expression in the Area of SCI

**FIGURE 5:**
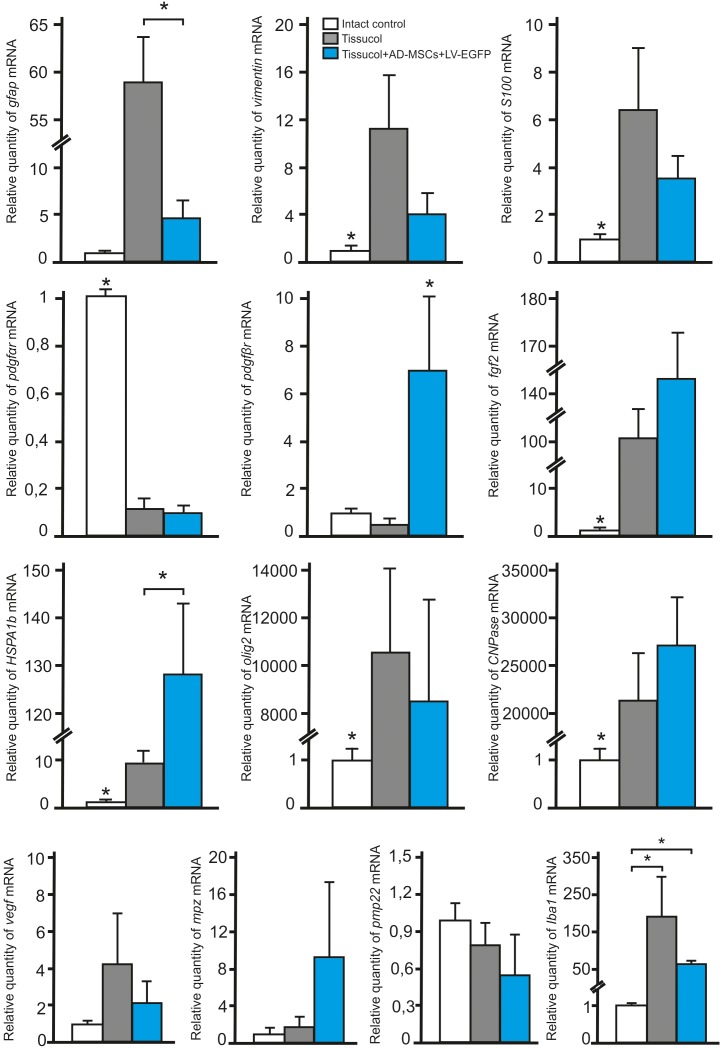
Analysis of changes in mRNAs expression in the area of SCI. GFAP, VIMENTIN, S100, PDGFαR, PDGFβR, FGF2, HSPA1b, OLIG2, CNPase, IBA1, MPZ, PMP22, and VEGF mRNA expression in intact spinal cord (write column) or in 60 days after Tissucol (gray column) and Tissucol+AD-MSCs+LV-EGFP (blue column) application. The mRNA expression levels in intact spinal cord was considered as 100%. ^∗^*P* < 0.05, one-way ANOVA followed by a Tukey’s *post hoc* test.

To investigate whether the application of AD-MSCs changes the expression of neural cells and neurotrophic factors mRNAs in injured spinal cord, qRT-PCR was performed on sections obtained from the injury site 60 days post-application of Tissucol. There was a statistically significant difference between the study groups in the mRNA expression levels of GFAP, VIMENTIN, S100, PDGFαR, PDGFβR, FGF2, HSPA1b, OLIG2, CNPase and IBA1, but not MPZ, PMP22, and VEGF (**Figure 5**).

In 60 days after Tissucol application (Tissucol+AD-MSCs+LV-EGFP and Tissucol groups) we observed a significantly increased expression of mRNA of GFAP, VIMENTIN, S100, FGF2, HSPA1b, OLIG2, CNPase, IBA1 and a decreased expression of PDGFαR mRNA, as compared to the intact controls (in all cases *P* < 0.05). Our results demonstrate that the mRNA expression of PDGFβR (*P* < 0.05) and HSPA1b (*P* < 0.05) were markedly upregulated, when compared to the Tissucol group and the intact controls. Nevertheless, the GFAP mRNA expression was markedly downregulated (*P* < 0.05) 60 days after application of Tissucol+AD-MSCs+LV-EGFP, as compared to the Tissucol group.

## Discussion

In this study we have evaluated structural and functional changes in the injured spinal cord in the setting of application of AD-MSCs mixed with the fibrin matrix Tissucol. The application of AD-MSCs was found to have a positive effect on the functional recovery after SCI that was confirmed by the results of behavioral and electrophysiological studies. It was previously demonstrated with the BBB test in the transected spinal cord model, that the use of neural stem cell- and Schwann cell-loaded biodegradable polymer scaffolds did not improve the locomotor function, though it supported axonal regeneration (Olson et al., 2009). However, other authors provided evidence of progressive functional recovery over time with stem cell-loaded biodegradable polymer scaffolds therapy after SCI in rodents (Hatami et al., 2009; Lu et al., 2012; Mothe et al., 2013). Our results obtained using the BBB test are consistent with those of the electrophysiological study. For example, on 60 day after the application of AD-MSCs we noted a better progress in the restoration of the H/M wave amplitude ratio that may reflect better progress in recovery after the spinal shock in this group. At the same time, we have not yet determined what caused a rise of the M-wave amplitude in the experimental group with AD-MSC application with fibrin matrix. It is possible that the application of AD-MSCs might lead to an increased number of muscle fibers. However, the morphometry of the gastrocnemius muscles did not detect any significant changes.

We also conducted a morphometric study to determine any difference in areas of the spared tissue and abnormal cavities between the experimental and control groups. On post-injury day 60 we found that the application of AD-MSCs resulted in a reduced cavity volume and improved tissue retention in the subacute phase following SCI. The ability of MSCs to improve tissue retention of the injured spinal cord has been previously demonstrated by other researchers (Ankeny et al., 2004; Bakshi et al., 2006; Urdzíková et al., 2006; Abrams et al., 2009; Zeng et al., 2011), however, not all studies confirm this potential (Yoshihara et al., 2006). The contradictory results in this case can be associated with different approaches to MSC transplantation, such as the timing of cell transplantation and their number, the routes of administration (intraspinal, intrathecal, intravenous, application), the presence of immunosuppression, as well as the quality of MSCs obtained in culturing and their secretome profile. We consider that the latter factor should be given particular attention. As there is greater variability among individual human/rodent donors, this could be reflected in the secretion profile of MSCs (Neuhuber et al., 2005; Yoshihara et al., 2006). Thus, one should consider the secretion profile of MSCs obtained, as the impact of their transplantation on maintenance of the nervous tissue structure within an area of damage is primarily associated with these cells expressing neurotrophic factors and cytokines by complex paracrine and autocrine mechanisms.

We have assessed the astroglial and microglial cells in this study. The results obtained confirm the evidence that AD-MSCs are able to prevent the second phase of neuronal injury by contributing to astroglia and microglia suppression (Ide et al., 2010; Laroni et al., 2015; Ribeiro et al., 2015). The latter is consistent with the results of Kim et al., which showed that intravenous injection of AD-MSCs after acute SCI in dogs may prevent further damage through enhancement of antioxidative and anti-inflammatory mechanisms including through lesser microglial infiltration (Kim et al., 2015). At the same time, using an immunofluorescence assay of the GFAP expression we detected that the application of AD-MSCs had a more effect on the reduction of astroglial activation cadually from the injury epicenter. The results obtained are consistent with the RT-PCR findings, where we found significant decreases in the expression of GFAP mRNA after application of Tissucol+AD-MSCs+LV-EGFP, as compared to the Tissucol group.

Our results demonstrate that mRNA expression of PDGFβR and HSPA1b was markedly upregulated after application of Tissucol+AD-MSCs+LV-EGFP, as compared to the Tissucol group and intact controls. The results obtained seem quite positive in the context of stimulating neuroregeneration. Thus, the increased expression of PDGFβR mRNA might indirectly indicate the stimulation of neovascularization (Betsholtz et al., 2005; Shaymardanova et al., 2012). In this aspect our data are consistent with the results obtained with transplantation of the MSCs encapsulated in a 3D gelatin scaffold with a thin layer of PLGA into the area of a spinal cord transection (Zeng et al., 2011). Simultaneously, PDGF stimulation may drive not only mitogenic activity of mesenchymal cells to regulate matrix metabolism, chemotactic and vasoactive properties, but also in parallel a fibrogenic stimulation. The latter is a particular consideration for liver damage to hepatic stellate cells (Pinzani et al., 1996; Friedman, 2000, 2008), but no attention has been paid in relation to nervous tissue. HSPA1b is a vital anti-apoptotic regulator, which can inhibit the processes of increasing intracellular calcium or Cytochrome C release. It was shown that its levels increased gradually and peaked at 1 day, but then returned to a level which was still higher than sham control 5 days after spinal cord contusion (Yan et al., 2010).

Thus, the study results suggest that the use of AD-MSCs combined with fibrin matrix as an application onto the site of SCI in a subacute period can affect the most important mechanisms of nerve tissue recovery. Considering their unique therapeutic properties, their ease of accessibility and expansion, AD-MSCs combined with a scaffold reveals a potential for a widespread use in clinical medicine. Nevertheless, there remain critical challenges – (1) standartization of generation protocols, including cell culture conditions, passage, and cell density, (2) the heterogeneity of secretory phenotype of the MSC population, (3) cellular mechanisms and biological properties of MSCs should be disclosed more clearly, (4) translation to the clinic will need preclinical studies on larger animals, (5) randomized, controlled, multicenter clinical trials are necessary to determine the optimal conditions and doses for MSC therapy.

## Author Contributions

YM: SCI and cell application, statistical analysis, and writing the article. EA: SCI and post-surgical care. AAK: behavioral study. EZ: isolation and preparation of rat mesenchymal stem cells. LG: histological assessment and immunohistochemical studies. EG: RNA isolation, cDNA synthesis, and real-time PCR. ARo: electrophysiological studies. APK and ARi: development of a research plan and participation in the writing of the article.

## Conflict of Interest Statement

The authors declare that the research was conducted in the absence of any commercial or financial relationships that could be construed as a potential conflict of interest.

## Footnotes

1 http://cels.uri.edu/gsc/cndna.html

2 syntol.ru

3 thermoscientific.com
